# Floral Induction of Longan (*Dimocarpus longan*) by Potassium Chlorate: Application, Mechanism, and Future Perspectives

**DOI:** 10.3389/fpls.2021.670587

**Published:** 2021-06-07

**Authors:** Shilian Huang, Dongmei Han, Jing Wang, Dongliang Guo, Jianguang Li

**Affiliations:** Key Laboratory of South Subtropical Fruit Biology and Genetic Resource Utilization (MOA), Guangdong Province Key Laboratary of Tropical and Subtropical Fruit Tree Research, Institute of Fruit Tree Research, Guangdong Academy of Agricultural Sciences, Guangzhou, China

**Keywords:** *Dimocarpus longan*, floral induction, KClO_3_, stress response, plant hormone

## Abstract

Longan (*Dimocarpus longan* L.) is one of the most important tropical and subtropical fruits in the world. Longan fruit has high nutritional and medical value, and is regarded as a treasure among fruits. Since it was first reported that potassium chlorate (KClO_3_) could be successfully applied to promote flowering in longan, this compound has been widely used in the production of on-season and off-season longan fruits. KClO_3_ has thus played a great role in promoting the development of the longan industry. In this review, we summarize the application methods, influencing factors, and physiological and molecular mechanisms associated with KClO_3_-mediated induction of longan flowering. It can be deduced that leaves may play a crucial role in the transport of and response to KClO_3_. Leaves supply carbon and nitrogen nutrition, and hormone and signaling molecules needed for the differentiation of apical buds. Moreover, cytokinins may be crucial for KClO_3_-mediated induction of longan flowering. More effort should be focused on studying the molecular mechanisms underlying this process. This will not only help us to better understand floral induction by KClO_3_ in longan but also enrich our understanding of flowering regulation mechanisms in woody plants.

## Introduction

Longan (*Dimocarpus longan* Lour.) is an important tropical and subtropical fruit tree that belongs to the Sapindaceae family (Lai et al., 2000). Native to southern China and Southeast Asia, longan is cultivated in more than 20 countries, from Sri Lanka and India to East Malaysia and Australia (Janick, 1989; Lithanatudom et al., 2017). China has the largest planting area and highest yield of longan, followed by Thailand, Vietnam, India, and South Africa. These five countries produce ninety percent longan (Food and Agriculture Organization, 2014; Wang et al., 2015). Longan can be eaten fresh or processed, and is widely consumed due to its sweet juicy taste and health benefits. These health benefits include blood metabolism promotion, memory enhancement and insomnia incidence reduction (Rangkadilok et al., 2007; Park et al., 2010). Longan have also been used as traditional Chinese medicine to treat leucorrhea, kidney disorders, allergies, and cardiovascular diseases (Jiang et al., 2009).

Floral induction (FI) is considered the first step from vegetative to reproductive growth. FI plays an important role as it determines the success of commercial orchards (Bangerth, 2009). The flowering of many plants is regulated by environmental factors, such as chilling, drought, and oxidative stresses. With the development of science and technology, it is possible to create similar environmental conditions for flowering requirement, or to find chemicals to promote off-season flowering. For example, supplementary lighting in short-day winters can induce flowering in long-day pitaya (*Hylocereus undatus* Britton et Rose) (Xiong et al., 2020). In addition, the use of paclobutrazol can induce off-season flowering in mango (*Mangifera indica* L.) (Sergent et al., 1997; Abdel Rahim et al., 2011).

Normally, a period of low temperature (usually ≤ 18°C) is required for longan to bloom (Suttitanawat et al., 2012). In warmer winter, girdling can control the growth of winter shoots and promote flowering in spring (Wu et al., 2000). In addition, low concentrations (100–200mg/L) of paclobutrazol (PP_333_) have been shown to significantly promote flower bud differentiation (Huang, 1996). The first report of potassium chlorate (KClO_3_) being used to induce flowering in longan was published by Changrui Yan. Since then, attempts have been made to achieve stable FI in longan (Yan et al., 1998; Li et al., 2006). Thus, KClO_3_ has now been widely used in longan production. Fresh longan fruits are currently available in local markets in Thailand all year round due to the application of KClO_3_ (Subhadrabandhu and Yapwattanaphun, 2000).

Thus far, FI by KClO_3_ has only been found to be effective for longan. Studying FI by KClO_3_ will provide a deeper understanding of the flowering regulation mechanism of woody plants. Furthermore, such study will provide a theoretical basis for regulating the flowering of other Sapindaceae plants, such as *Litchi chinensis* Sonn. In this paper, we summarize the application methods, physiological and molecular regulation mechanisms, influencing factors, and the environmental impact of KClO_3_-mediated FI in longan. Furthermore, the future perspectives of studying the mechanisms underlying FI by KClO_3_ are analyzed.

## Application Methods

Year-round FI is achieved in longan with KClO_3_ application. The perfect time period to apply KClO_3_ for on-season FI is from November to January, after the maturation of the last shoot. Off-season FI can be achieved by applying KClO_3_ from February to October, when the leaves of the last shoot are light green. Leaves play a fundamental and essential role in this process of FI (Nunez-Elisea et al., 1996). To achieve higher FI efficiency, KClO_3_ should be applied when leaves are older than 60 days, as flower bud differentiation requires adequate nutrition. It has been proposed by Hegele et al. (2004) that the presence of young leaves reduces the efficiency of FI by KClO_3_. Furthermore, supernutrition can lead to flushes of new leaves, and thus the timing of KClO_3_ treatment needs to be precisely controlled (Lu et al., 2006).

Foliar spraying and soil drenching are the most common KClO_3_ application approaches. For foliar spraying, 0.5–3 g/L KClO_3_ solution is sprayed onto the leaves. For soil drenching, a circular shallow ditch with a depth of approximately 15–20 cm and a width of approximately 15–25 cm should be dug along the drip line of the longan tree crown. Then, 0.5–2 kg of solid KClO_3_ or water solution should be spread into the ditch. Generally, the combination of the two methods will produce better results. The specific KClO_3_ dosage should be determined according to the plant variety and age, and to the climate. In addition, the soil needs to be kept slightly wet for 15 days after KClO_3_ treatment. Furthermore, fertilization and pruning should be avoided during the period between the treatment and flowering (Huang et al., 2009).

## Factors Influencing KCLO_3_-Mediated FI

Floral induction is highly correlated with leaf age in tropical and subtropical fruit trees. Longan apical buds with high carbon content (> 50.93 mg/g) in the leaves can be induced into flowers. Meanwhile, those with leaves with low carbon content (< 37.40 mg/g) cannot be induced by KClO_3_ out of season (Hong et al., 2014). Previous research has suggested that mature leaves might be involved in the conversion of isopentenyladenine/isopentenyladenosine (iP/iPA) cytokinin (CK) precursors into the active zeatin/zeatin riboside (Z/ZR) CKs. Hence, leaves may be involved in the FI process (Potchanasin et al., 2009a; Tiyayon et al., 2010). Shading has been shown to inhibit the export of indoleacetic acid (IAA) out of the shoot apical buds, the leaf export of iP/iPA- and Z/ZR-type CKs, and CK accumulation in shoot apical buds. In this way, shading can prevent FI by KClO_3_ treatment (Sritontip et al., 2008; Sringarm et al., 2009a; Ongprasert et al., 2010). Appropriate KClO_3_ concentrations promote flowering, while excessive KClO_3_ concentrations may lead to less flowering, no flowering or leaf burn (Ongprasert et al., 2010). Weather conditions can also affect KClO_3_-mediated FI; the lowest flowering percentage (11.9%–50.9%) occurs in rainy seasons, and higher flowering percentage (77.5–88.6%) in cool and hot seasons (Manochai et al., 2005).

## Absorption and Metabolism of Chlorate

**FIGURE 1:**
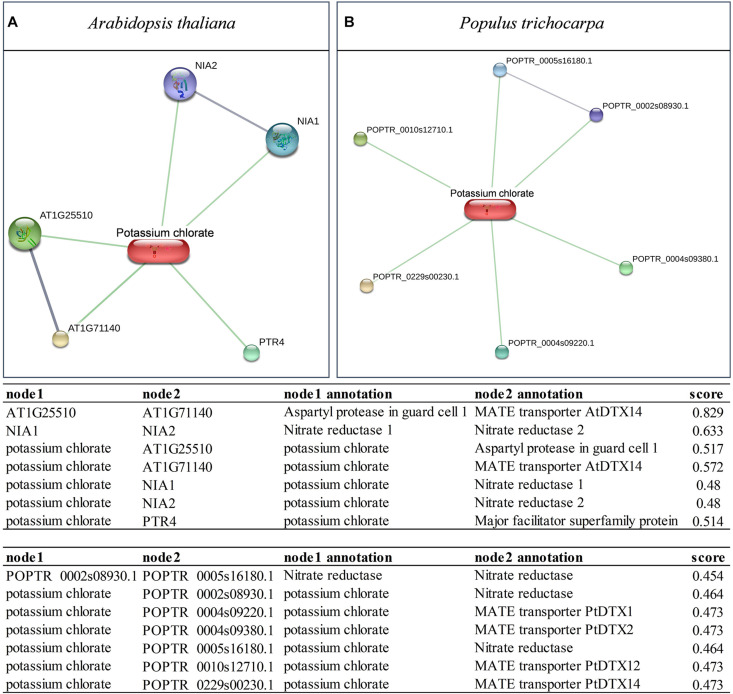
Interactions of potassium chlorate and proteins in *Arabidopsis thaliana* **(A)** and *Populus trichocarpa* **(B)** searched by STITCH 5.0. **(A)** Generated by input “Item Name: potassium chlorate, Organism: *Arabidopsis thaliana*”; **(B)** Generated by input “Item Name: potassium chlorate, Organism: *Populus trichocarpa.*” Stronger associations are represented by thicker lines and higher scores in the follow table. Protein-protein interactions are shown in gray and chemical-protein interactions in green.

Plant cells share the same absorption mechanism for chlorate and nitrate through nitrate transporter (Glass et al., 1999). Chlorate is generally not toxic to plants; however, chlorate becomes toxic when converted into chlorite and hypochlorite by nitrate reductase (NR) and nitrite reductase (NiR) (Hofstra, 1977). Chlorate is not harmful while plants lack NR activity (Doddema et al., 1978; Borges et al., 2004). The interactions of potassium chlorate and proteins in *Arabidopsis thaliana* and *Populus trichocarpa* were searched by STITCH 5.0 at http://stitch.embl.de/cgi/ (Szklarczyk et al., 2016). The interaction of two NRs with potassium chlorate was found in *Arabidopsis thaliana* and *Populus trichocarpa* (Figure 1). The following were also observed: one multidrug and toxic compound extrusion (MATE) transporter, one major facilitator superfamily transporter, one aspartyl protease in guard cell 1 in *Arabidopsis thaliana* (Figure 1). Meanwhile, four MATE transporters were found in *Populus trichocarpa* (Figure 1). These findings indicate that nitrate reduction systems are crucial in chlorate absorption and metabolism. MATE may pump chlorate out of cells or transfer chlorate into vacuoles for detoxification due to its implication in the membrane-mediated transport of small organic molecules, metal ions, and chloride ions (Zhang et al., 2017; Upadhyay et al., 2019).

## Physiological Mechanisms of KCLO_3_-Mediated FI

### Plant Hormones

Endogenous plant hormones participate in the entire life process of plants. Plant hormones regulate plant growth and development by forming a complex and complete signaling network that enables the transmission of exogenous or endogenous signals. Therefore, hormone signals are crucial to flower formation (Santner and Estelle, 2009; Wolters and Jürgens, 2009; Puja et al., 2015). Application of KClO_3_ can induce changes in endogenous hormones. The contents of two types of CK—trans-zeatin (tZ)/ZR and iP/iPA—have been shown to increase after KClO_3_ treatment in apical buds, sub-apical wood and bark, leaves and roots (Potchanasin et al., 2009b; Hegele et al., 2010; Suttitanawat et al., 2012). However, only ZR seemed to be involved in the FI process (Bangerth et al., 2010). Mature leaves may set the stage for the conversion of iPA into ZR, and KClO_3_ treatment promotes the translocation of CKs from the leaves to apical buds (Sringarm et al., 2009b; Tiyayon et al., 2010). The increased CK contents can also be detected during flowering in longan (Sringarm et al., 2009b). The contents of gibberellic acid (GA), IAA, and abscisic acid (ABA) in shoot apical buds and leaves have been shown to decrease following KClO_3_ application (Hegele et al., 2008, 2010; Tiyayon et al., 2010). This shows that longan FI by KClO_3_ may require more CKs, but less GA, IAA, and ABA. Increased production of ethylene (ETH) has also been detected in longan leaves after KClO_3_ treatment. It is unknown whether this increase is involved in the FI process or a stress response (Sringarm et al., 2009b). Besides ETH and CK, KClO_3_ treatment can also increase the amount of salicylic acid (SA). SA has also been found to be closely related to plant flower formation (Sringarm et al., 2009b; Martínez et al., 2010).

### Carbon and Nitrogen Nutrition

The carbon–nitrogen (C:N) ratio is an important physiological factor influencing flowering. Carbohydrate reserves are a prerequisite for FI in tropical and subtropical trees. High carbohydrate and low nitrogen contents lead to a high C:N ratio, which is conducive to flowering. Meanwhile, a high nitrogen content results in a low C:N ratio, which is favorable for vegetative growth (Corbesier et al., 2002). It seems that KClO_3_ treatment does not impact the total nitrogen content, total non-structural carbohydrate content, or carbohydrate–nitrogen ratio (Charoensri et al., 2005; Wangsin and Pankasemsuk, 2005; Matsumoto et al., 2007). However, it has been shown that the content of soluble sugar, fructose, and glucose increased, the sucrose content increased significantly, and the starch content decreased significantly in longan leaves in response to KClO_3_ treatment (Chen and Li, 2004; Lu, 2005; Chang, 2010). KClO_3_ treatment has also been shown to reduce the longan leaf nitrate reductase activity. In the same study, KClO_3_ led to a peak in soluble amino acid accumulation in the leaves within 2 weeks of treatment; this amino acid accumulation then decreased but continued to increase in the apical buds (Lu, 2005). Generally, higher ammoniacal nitrogen contents are beneficial for flowering. Overall, it can be inferred that KClO_3_ treatment can change the types of carbohydrate and protein to promote reproductive development without affecting total nitrogen or total non-structural carbohydrate contents.

### Stress Response

KClO_3_ is a type of strong oxidant that is used as a herbicide. High KClO_3_ concentrations will cause longan leaves to turn yellow and fall off. It is speculated that appropriate amounts of KClO_3_ can lead to stress responses. Reduced net carbon dioxide (CO_2_) assimilation, transpiration, stomatal conductance rates, and photosystem II efficiencies (*F*_*v*_/*F*_*m*_) have been detected after KClO_3_ treatment (Hegele et al., 2008; Sritontip et al., 2010, 2013). The treatment has resulted in chlorophyll degradation, the destruction of chloroplast thylakoid membrane structure, the disappearance of chloroplast starch granules, and the destruction of basal granules (Lu, 2005). The decrease in photosynthetic capacity has been shown to have been mainly caused by the inhibition of the activity of the photosynthetic apparatus (Chang, 2010). The reactive oxygen species and malondialdehyde contents, and superoxide dismutase and peroxidase activities, were found to be higher in leaves within 1 month after KClO_3_ treatment than in the control. Meanwhile, the leaf water potential and root activity were significantly lower than in the control (Ouyang et al., 2005).

## Molecular Mechanisms of KCLO_3_-Mediated FI

Several genes related to the flowering of longan have been identified. Tiyayon et al. (2011) first cloned the longan *flowering locus T* (*DlFT*) gene, which shared 68% identity with the *Arabidopsis thaliana* gene, *AtFT* (Tiyayon et al., 2011). Winterhagen et al. (2013) isolated *DlFT1*, *DlFT2*, and two *APETALA1*-like (*DlAP1-1* and *DlAP1-2*) sequences from longan. Transgenic analysis indicated that *DlFT1* promoted flowering, while *DlFT2* inhibited flowering. Ectopic overexpression of *AP1* genes in *Arabidopsis* resulted in early or late-flowering phenotypes (Winterhagen et al., 2013). Overexpression of the longan *gigentea* (*DlGI*) and *flavin-binding*, *kelch repeat*, *F-box 1* (*DlFKF1*) genes caused *Arabidopsis* to bloom early under long-day conditions (Huang et al., 2017). The early flowering 4 proteins, DlELF4-1 and DlELF4-2, were found to bind to and activate the promoter of *DlGI* (Waheed et al., 2020). Through transcriptome analysis of “Sijimi” longan, Zhang et al. (2016) found a large number of genes related to the four known flowering pathways and floral integrator genes. By comparing and analyzing the different expression levels of genes in the terminal tips of “Sijimi” and “Lidongbe” longan, *short vegetative phase* (*SVP*), *GI*, *FKF1*, and *ELF4* were found to be involved in the continuous flowering of “Sijimi,” and *ELF4* might play a key role.

Sixty-five uniquely expressed genes were identified between buds with and without KClO_3_ treatment, and many of them were demonstrated to be involved in shoot and floral meristem development. These genes included homologs of *protodermal factor 1* (*PDF1*), *SHEPHERD*, and *PISTILLATA* (Matsumoto, 2006; Matsumoto et al., 2007). KClO_3_ treatment was also found to enhance the expression of *DlFT1* in mature leaves, which was highly consistent with the increased CK content (Winterhagen et al., 2020).

## Conclusion

**FIGURE 2:**
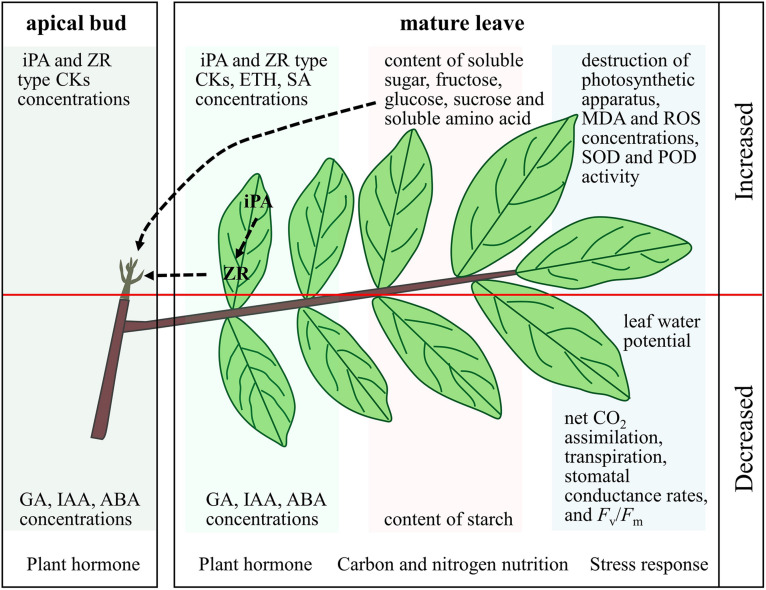
Physiological mechanism of floral induction by KClO_3_ in longan. The physiological indexes above the red line increased after KClO_3_ treatment, while the indexes under the red line decreased.

By analyzing the results of previous studies, it can be inferred that in longan, leaves are the main plant organs that respond to KClO_3_ treatment. KClO_3_ treatment can induce stress responses in leaves. These stress responses include reduced leaf water potential, net CO_2_ assimilation, transpiration, stomatal conductance rates and *F*_*v*_/*F*_*m*_, increased destruction of photosynthetic apparatus, malondialdehyde and reactive oxygen species contents, and superoxide dismutase and peroxidase activities (Figure 2; Ouyang et al., 2005; Sritontip et al., 2013). KClO_3_ treatment can also cause changes in carbon and nitrogen nutrition in longan leaves. It reduces the starch content and increases the soluble sugar, fructose, glucose, sucrose, and soluble amino acid contents (Figure 2; Lu, 2005; Chang, 2010). Furthermore, KClO_3_ treatment can lead to changes in leaf hormone contents. The treatment slightly reduces the contents of GA, IAA, and ABA and increases the contents of ETH, SA, and CKs (Figure 2; Sringarm et al., 2009b; Martínez et al., 2010). CKs may play a particularly vital role in FI by KClO_3_. KClO_3_ treatment can also slightly reduce the contents of GA, IAA, and ABA, and increase the contents of iPA- and ZR-type CKs in the apical bud, which may be due to transport from the leaves (Figure 2; Sringarm et al., 2009b; Tiyayon et al., 2010). The enhanced soluble sugar and soluble amino acid contents provide nutrition for flower bud differentiation. In addition, the H_2_O_2_ generated in the stress response process may act as an important signal molecule in off-season FI of longan as it can promote the expression of *DlAP1* and *DlFT* (Hong et al., 2015; Yang et al., 2016).

**FIGURE 3:**
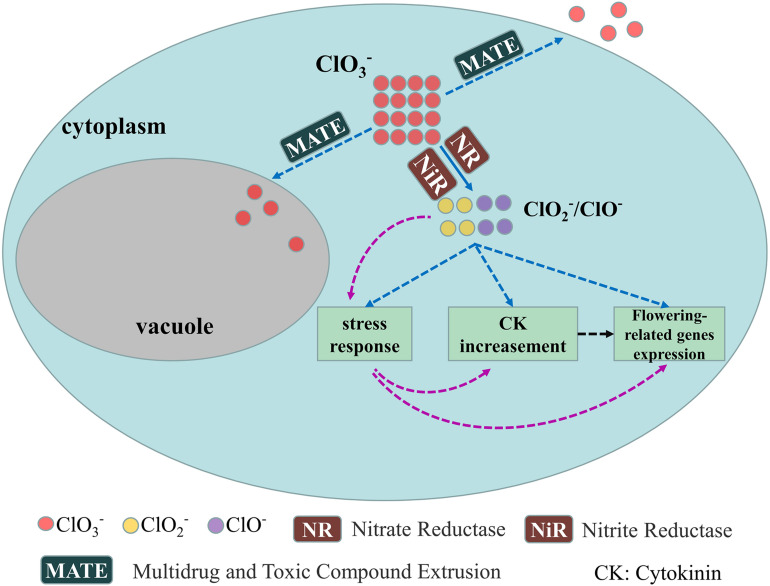
Proposed model of the molecular regulation of KClO_3_ mediated FI. The solid line shows confirmed result while dotted lines indicate speculated. Blue dotted lines (after ClO_2_^–^/ClO^–^): ClO_2_^–^/ClO^–^ may directly induce stress response, CK content increasement and flowering-related genes expression; Red dotted lines: ClO_2_^–^/ClO^–^ induce stress response, then stress response induce CK content increasement and flowering-related genes expression; Black dotted line: CK content increasement may also induce flowering-related genes expression.

We have a general understanding of the physiological mechanism underlying longan FI by KClO_3_. Though little is known about the corresponding molecular mechanism, a speculated regulation model of KClO_3_-mediated FI was proposed based on above information. Chlorate can be reduced to chlorite and hypochlorite by nitrate reductase (NR) and nitrite reductase (NiR) (Figure 3; Borges et al., 2004). Chlorite and hypochlorite may directly cause stress response, CK content increasement and expression of flowering-related genes (Figure 3, blue dotted lines). It is reported that stress could induce the cytokinin synthesis (Reguera et al., 2013) and flowering (Cho et al., 2017). There is another possibility that the stress response caused by chlorite and hypochlorite may contribute to the CK content increasement and expression of flowering-related genes (Figure 3, red dotted lines). Also, the enhanced CK content may induce the expression of flowering related genes as indicated by Winterhagen et al. (2020) (Figure 3, black dotted lines). Excess chlorate in plant cell may be transferred out of cell or into vacuole by MATE transporters (Figure 3, blue dotted lines).

It remains to be understood how KClO_3_ is transferred from the root to leaf or from the leaf to root. Furthermore, it is unclear how KClO_3_ causes various physiological changes and why it is possible to use KClO_3_ in place of cold treatment in FI. In particular, it will be important to understand the relationship between KClO_3_ treatment and the contents of CKs, and what role CKs play in FI by KClO_3_. Addressing these issues will not only provide a full understanding of the mechanism underlying FI by KClO_3_ in longan but also enrich our theoretical understanding of flowering regulation in woody plants. Although the genome of longan has been published (Lin et al., 2017), a genetic transformation system, or a highly efficient virus-induced gene silencing system for longan, will be required to undertake the necessary further research.

## Author Contributions

SH, DH, and JW wrote the manuscript. SH initiated the idea of the review. DG and JL revised the manuscript. All authors contributed to the article and approved the submitted version.

## Conflict of Interest

The authors declare that the research was conducted in the absence of any commercial or financial relationships that could be construed as a potential conflict of interest.

## References

[B1] Abdel Rahim A. O. S. Elamin O. M. Bangerth F. K. (2011). Effects of paclobutrazol (PBZ) on floral induction and associated hormonal and metabolic changes of biennially bearing mango (*Mangifera indica* L.) cultivars during off year. *J. Agr. Bio. Sci.* 6 55–67.

[B2] Bangerth K. F. (2009). Floral induction in mature, perennial angiosperm fruit trees: similarities and discrepancies with annual/biennial plants and the involvement of plant hormones. *Sci. Hortic.* 122 153–163. 10.1016/j.scienta.2009.06.014

[B3] Bangerth K. F. Potchanasin P. Sringarm K. Dongliang Q. Mitra S. K. Diczbalis Y. (2010). Hormonal regulation of the regular and ‘off season’ floral induction process of longan (*Dimocarpus longan*) trees. *Acta Hortic.* 863 215–224. 10.17660/ActaHortic.2010.863.28

[B4] Borges R. Miguel E. C. Dias J. M. D. da Cunha M. Bressan-Smith R. E. de Oliveira J. G. (2004). Ultrastructural, physiological and biochemical analyses of chlorate toxicity on rice seedlings. *Plant Sci.* 166 1057–1062. 10.1016/j.plantsci.2003.12.023

[B5] Chang Q. (2010). *Study on relationship between off-season floral induction in longan and nutrition of the carbon and nitrogen*, Master’s thesis, Fuzhou: Fujian Agriculture & Forestry University.

[B6] Charoensri P. Jutamanee K. Tawatpun J. Tongumpai P. Krisanapook K. (2005). Effects of potassium chlorate and girdling on flowering of ‘Phet Sakhon’ longan. *Acta Hortic*. 665 259–268. 10.17660/ActaHortic.2005.665.30

[B7] Chen Q. X. Li S. G. (2004). KClO_3_ induced longan to form floral bud and bloom and its changes of carbohydrates and protein in leaves. *J. Fujian Agr. Forestry U* 33 182–185.

[B8] Cho L. H. Yoon J. An G. (2017). The control of flowering time by environmental factors. *Plant J.* 90 708–719. 10.1111/tpj.13461 27995671

[B9] Corbesier L. Bernier G. Périlleux C. (2002). C:N ratio increases in the phloem sap during floral transition of the long-day plants *Sinapis alba* and *Arabidopsis thailiana*. *Plant Cell Physiol.* 43 684–688. 10.1093/pcp/pcf071 12091723

[B10] Doddema H. Hofstra J. J. Feenstra W. J. (1978). Uptake of nitrate by mutants of *Arabidopsis thaliana*, disturbed in uptake or reduction of nitrate: I. effect of nitrogen source during growth on uptake of nitrate and chlorate. *Physiol. Plant.* 43 343–350. 10.1111/j.1399-3054.1978.tb01592.x

[B11] Food and Agriculture Organization (2014). *Tropical fruits compendium.* Available online at: http://www.fao.org/docrep/meeting/022/am481t.pdf (accessed September 20, 2014).

[B12] Glass A. D. M. Erner Y. Hunt T. Kronzucker H. J. Okamoto M. Rawat S. (1999). “Inorganic nitrogen absorption by plant roots,” in *Plant Nutrition-Molecular Biology and Genetics*, Gissel-Nielsen, G., Jensen, A (Dordrecht: Springer), 1–16. 10.1007/978-94-017-2685-6_1

[B13] Hegele M. Manochai P. Naphrom D. Sruamsiri P. Wünsche J. (2008). Flowering in longan (*Dimocarpus longan* L.) induced by hormonal changes following KClO_3_ applications. *Europ. J. Hort. Sci.* 73 49–54.

[B14] Hegele M. Naphrom D. Manochai P. Chattrakul A. Sruamsiri P. Bangerth F. (2004). Effect of leaf age on the response of flower induction and related hormonal changes in longan trees after KClO_3_ treatment. *Acta Hortic.* 653 41–49. 10.17660/ActaHortic.2004.653.4

[B15] Hegele M. Sritontip C. Chattrakul A. Tiyayon P. Wünsche J. N. (2010). Hormonal control of flower induction in litchi and longan. *Acta Hortic.* 863 305–314. 10.17660/ActaHortic.2010.863.40

[B16] Hong J. Chen X. Li S. Zhang L. Yang Z. (2015). The impact of H_2_O_2_ and NO on *FT* gene expression and floral bud formation in longan. *Chin. J. Trop. Crop*. 36 2002–2006.

[B17] Hong J. W. Li S. G. Zhang L. Yang Z. Q. (2014). Carbon analysis on flowering differentiation in off-season longan. *Guangdong Agr. Sci.* 151 921–928. 10.16768/j.issn.1004-874x.2014.16.030

[B18] Hofstra J. J. (1977). Chlorate toxicity and nitrate reductase activity in tomato plants. *Physiol. Plant* 41 65–69. 10.1111/j.1399-3054.1977.tb01524.x

[B19] Huang F. Fu Z. Zeng L. Morley-Bunker M. (2017). Isolation and characterization of *GI* and *FKF1* homologous genes in the subtropical fruit tree *Dimocarpus longan*. *Mol. Breeding* 37:90. 10.1007/s11032-017-0691-z

[B20] Huang J. Y. Pang X. H. Zhou Q. G. (2009). Research on the technology of forcing logan. *J. Guangxi Vocational. Tech. Coll.* 2 5–6.

[B21] Huang Q. W. (1996). Effects of plant growth regulators on endogenous hormones and bud differentiation of longan. *Acta Bot. Yunnanica* 18 145–150.

[B22] Janick J. (1989). *Horticultural Reviews.* Portland: Timber Press, 10.1002/9781118060841

[B23] Jiang G. Jiang Y. Yang B. Yu C. Tsao R. Zhang H. (2009). Structural characteristics and antioxidant activities of oligosaccharides from longan fruit pericarp. *J. Agric. Food Chem.* 57 9293–9298. 10.1021/jf902534v 19739643

[B24] Lai Z. Chen C. Zeng L. Chen Z. (2000). “Somatic embryogenesis in longan [*Dimocarpus longan* Lour.],” in *Somatic Embryogenesis in Woody Plants*, eds Jain S. M. Gupta P. Newton R. (Netherlands: Springer), 415–431. 10.1007/978-94-017-3030-3_13

[B25] Li M. Ren L. Wang Y. (2006). Review on applications and mechanism of KClO_3_ on inducing flower formation of Longan (*Dimocarpus longan* Lour.) trees. *J. South. Agr.* 37 293–297.

[B26] Lin Y. L. Min J. M. Lai R. L. Wu Z. Y. Chen Y. K. Yu L. L. (2017). Genome-wide sequencing of longan (*Dimocarpus longan* lour.) provides insights into molecular basis of its polyphenol-rich characteristics. *GigaScience* 6 1–14. 10.1093/gigascience/gix023 28368449 PMC5467034

[B27] Lithanatudom S. K. Chaowasku T. Nantarat N. Jaroenkit T. Smith D. R. Lithanatudom P. (2017). A first phylogeny of the genus *Dimocarpus* and suggestions for revision of some taxa based on molecular and morphological evidence. *Sci. Rep.* 7:6716. 10.1038/s41598-017-07045-7 28751754 PMC5532229

[B28] Lu J. M. (2005). *A physiological study on biological effects of potassium chlorate on longan (Dimocarpus longan* Lour.), master’s thesis, Guangzhou: South China Agricultural University.

[B29] Lu J. M. Huang X. M. Wang H. C. Zhang C. L. Yang R. T. Xie L. (2006). Current situation in the research and application of chlorate in longan production and approaches to understanding the mechanisms of the biological effects of chlorate. *Plant Physiol. J.* 42 567–572.

[B30] Manochai P. Sruamsiri P. Wiriya-Alongkorn W. Naphrom D. Hegele M. Bangerth F. (2005). Year around off-season flower induction in longan (*Dimocarpus longan* Lour.) trees by KClO_3_ applications: potentials and problems. *Sci. Hortic*. 104 379–390. 10.1016/j.scienta.2005.01.004

[B31] Martínez C. Pons E. Prats G. León J. (2010). Salicylic acid regulates flowering time and links defence responses and reproductive development. *Plant J.* 37 209–217. 10.1046/j.1365-313X.2003.01954.x 14690505

[B32] Matsumoto T. K. (2006). Genes uniquely expressed in vegetative and potassium chlorate induced floral buds of *Dimocarpus longan*. *Plant Sci.* 170 500–510. 10.1016/j.plantsci.2005.09.016

[B33] Matsumoto T. K. Tsumura T. Zee F. (2007). Exploring the mechanism of potassium chlorate-induced flowering in *Dimocarpus longan*. *Acta Hortic*. 738 451–458. 10.17660/ActaHortic.2007.738.56

[B34] Nunez-Elisea E. R. Davenport T. L. Carderia M. L. (1996). Control of bud morphogenesis in mango (*Mangifera indica* L.) by girdling, defoliation and temperature modification. *J. Hort. Sci*. 71 25–39. 10.1080/14620316.1996.11515379

[B35] Ongprasert S. Wiriya-Alongkorn W. Spreer W. (2010). The factors affecting longan flower induction by chlorate. *Acta horticulturae* 863 375–380. 10.17660/actahortic.2010.863.50

[B36] Ouyang R. Liu H. P. Li P. Wang H. C. Hu G. B. (2005). Physiological responses of longan to KClO_3_ stress. *Acta Agr. U. Jiangxiensi* 27 34–38.

[B37] Park S. J. Dong H. P. Dong H. K. Lee S. Yoon B. H. Jung W. Y. (2010). The memory-enhancing effects of *Euphoria longan* fruit extract in mice. *J. Ethnopharmacol.* 128 160–165. 10.1016/j.jep.2010.01.001 20064595

[B38] Potchanasin P. Sringarm K. Naphrom D. Bangerth K. F. (2009a). Floral induction in longan (*Dimocarpus longan*, Lour.) trees: iv. the essentiality of mature leaves for potassium chlorate induced floral induction and associated hormonal changes. *Sci. Hortic.* 122 312–317. 10.1016/j.scienta.2009.06.007

[B39] Potchanasin P. Sringarm K. Sruamsiri P. Bangerth K. F. (2009b). Floral induction (FI) in longan (Dimocarpus longan Lour.) trees: part i. low temperature and potassium chlorate effects on fi and hormonal changes exerted in terminal buds and sub-apical tissue. *Sci. Hortic.* 122 288–294. 10.1016/j.scienta.2009.06.008

[B40] Puja O. Renu B. Shagun B. Ravinderjit K. Shivam J. Anjali K. (2015). The Common Molecular Players in Plant Hormone Crosstalk and Signaling. *Curr. Protein Pept. Sc*. 16 369–388. 10.2174/1389203716666150330141922 25824391

[B41] Rangkadilok N. Sitthimonchai S. Worasuttayangkurn L. Mahidol C. Ruchirawat M. Satayavivad J. (2007). Evaluation of free radical scavenging and antityrosinase activities of standardized longan fruit extract. *Food Chem. Toxicol.* 45 328–336. 10.1016/j.fct.2006.08.022 17049706

[B42] Reguera M. Peleg Z. Abdel-Tawab Y. M. Tumimbang E. B. Delatorre C. A. Blumwald E. (2013). Stress-induced cytokinin synthesis increases drought tolerance through the coordinated regulation of carbon and nitrogen assimilation in rice. *Plant Physiol.* 163 1609–1622. 10.1104/pp.113.227702 24101772 PMC3850209

[B43] Santner A. Estelle M. (2009). Recent advances and emerging trends in plant hormone signalling. *Nature* 459 1071–1078. 10.1038/nature08122 19553990

[B44] Sergent E. Ferrari D. Leal F. (1997). Effects of potasium nitrate and paclobutrazol on flowering induction and yield of mango (*Mangifera indica* L.) CV. *Haden. Acta Hortic.* 455 180–187. 10.17660/ActaHortic.1997.455.25

[B45] Sringarm K. Potchanasin P. Naphrom D. Bangerth K. F. (2009a). Floral induction (FI) in longan (*Dimocarpus longan* Lour.) trees. iii: effect of shading the trees on potassium chlorate induced FI and resulting hormonal changes in leaves and shoots. *Sci. Hortic.* 122 301–311. 10.1016/j.scienta.2009.06.006

[B46] Sringarm K. Potchanasin P. Sruamsiri P. Bangerth K. F. (2009b). Floral induction (FI) in longan (*Dimocrapus longan* Lour.) trees – The possible participation of endogenous hormones: II. Low temperature and potassium chlorate effects on hormone concentration in and their export out of leaves. *Sci. Hortic.* 122 295–300. 10.1016/j.scienta.2008.11.031

[B47] Sritontip C. Khaosumain Y. Changjeraja S. Changjeraja R. (2008). Effects of light intensity and potassium chlorate on photosynthesis and flowering in ‘Do’ Longan. *Acta Hortic*. 787 285–288. 10.17660/actahortic.2008.787.33

[B48] Sritontip C. Tiyayon P. Hegele M. Sruamsiri P. Wünsche J. N. (2010). Effects of temperature and potassium chlorate on leaf gas exchange and flowering in longan. *Acta Hortic.* 663 323–328. 10.17660/actahortic.2010.863.42

[B49] Sritontip C. Tiyayon P. Sringam K. Pantachod S. Naphrom D. Ruamrungsri S. (2013). Influence of water regimes and potassium chlorate on floral induction, leaf photosynthesis and leaf water potential in longan. *J. Agr. Sci*. 5 211–220. 10.5539/jas.v5n6p211

[B50] Subhadrabandhu S. Yapwattanaphun C. (2000). Regulation of flowering time for longan (*Dimorcarpus longan*) production in Thailand. *J. Appl. Horticult.* 2 102–105. 10.37855/jah.2000.v02i02.31

[B51] Suttitanawat P. Sruamsiri P. Sringarm K. (2012). Changes in cytokinins concentrations during induction period of longan cv. Daw in sand culture. *J. Agr. Tech.* 8 2353–2362.

[B52] Szklarczyk D. Santos A. von Mering C. Jensen L. J. Bork P. Kuhn M. (2016). STITCH 5: augmenting protein-chemical interaction networks with tissue and affinity data. *Nucleic Acids Res.* 44 D380–D384. 10.1093/nar/gkv1277 26590256 PMC4702904

[B53] Tiyayon P. Hegele M. Wünsche J. N. Pongsriwat K. Sruamsiri P. Samach A. (2011). Studies on the molecular basis of flowering in longan (*Dimocarpus longan*). *Acta Hortic*. 903 979–985. 10.17660/actahortic.2011.903.137

[B54] Tiyayon P. Sritontip C. Hegele M. Manochai P. Sruamsiri P. Wünsche J. N. (2010). Effects of girdling and defoliation on hormonal changes during flower induction in longan (Dimocarpus longan lour.). *Acta Hortic.* 863 329–334. 10.17660/actahortic.2010.863.43

[B55] Upadhyay N. Kar D. Mahajan D. B. Nanda S. Rahiman R. Panchakshari N. (2019). The multitasking abilities of MATE transporters in plants. *J. Exp. Bot*. 70 4643–4656. 10.1093/jxb/erz246 31106838

[B56] Waheed S. Peng Y. Zeng L. (2020). Identification and characterization of *DlGI* promoter involved in photoperiod, light intensity, hormone, and DlELF4 response from longan. *J. Am. Soc. Hortic. Sci.* 145 340–348. 10.21273/JASHS04946-20

[B57] Wang B. Tan H. W. Fang W. Meinhardt L. W. Mischke S. Matsumoto T. (2015). Developing single nucleotide polymorphism (SNP) markers from transcriptome sequences for identification of longan (*Dimocarpus longan*) germplasm. *Hortic. Res.* 2:14065. 10.1038/hortres.2014.65 26504559 PMC4595986

[B58] Wangsin N. Pankasemsuk T. (2005). Effect of potassium chlorate on flowering, total nitrogen, total nonstructural carbohydrate, C/N ratio, and contents of cytokinin-like and gibberellin-like substances in stem apex of ‘do’ longan. *Acta Hortic.* 665 255–258. 10.17660/ActaHortic.2005.665.29

[B59] Winterhagen P. Hegele M. Tiyayon P. Wünsche J. N. (2020). Cytokinin accumulation and flowering gene expression are orchestrated for floral meristem development in longan (*Dimocarpus longan* Lour.) after chemical flower induction. *Sci. Hortic.* 270:109467. 10.1016/j.scienta.2020.109467

[B60] Winterhagen P. Tiyayon P. Samach A. Hegele M. Wünsche J. N. (2013). Isolation and characterization of *FLOWERING LOCUS T* subforms and *APETALA1* of the subtropical fruit tree *Dimocarpus longan*. *Plant Physiol. Biochem*. 71 184–190. 10.1016/j.plaphy.2013.07.013 23954797

[B61] Wolters H. Jürgens G. (2009). Survival of the flexible: hormonal growth control and adaptation in plant development. *Nat. Rev. Genet.* 10 305–317. 10.1038/nrg2558 19360022

[B62] Wu D. Y. Qiu J. D. Zhang H. L. Luo X. Z. (2000). A study on flowering promotion by ringing in longan (*Dimocarpus longan* Lour.). *Sci. Agr. Sinica* 33 40–43.

[B63] Xiong R. Liu C. Xu M. Wei S. S. Huang J. Q. (2020). Transcriptomic analysis of flower induction for long-day pitaya by supplementary lighting in short-day winter season. *BMC Genomics* 21:329. 10.1186/s12864-020-6726-6 32349680 PMC7191803

[B64] Yan C. R. Zhao Z. N. Zhang Z. W. (1998). Effects of chemical on introducing blossom of longan. *J. Chinese Soc. Hort. Sci.* 44 517–518.

[B65] Yang Z. Xu Z. Zhang L. Hong J. Chen Y. Li S. (2016). Effect of H_2_O_2_ and NO on *AP1* gene expression and out of season flower bud formation in longan. *South China Fruits* 45 13–17.

[B66] Zhang H. Zhao F. G. Tang R. J. Yu Y. Song J. Wang Y. (2017). Two tonoplast MATE proteins function as turgor-regulating chloride channels in Arabidopsis. *Proc. Natl. Acad. Sci. USA* 114 E2036–E2045. 10.1073/pnas.1616203114 28202726 PMC5347570

[B67] Zhang H. N. Shi S. Y. Li W. C. Shu B. Liu L. Q. Xie J. H. (2016). Transcriptome analysis of ‘Sijihua’ longan (*Dimocarpus longan* L.) based on next-generation sequencing technology. *J. Hortic. Sci. Biotechnol.* 91 180–188. 10.1080/14620316.2015.1133539

